# Evaluation of the influence of pulmonary hypertension in ultra-fast-track anesthesia technique in adult patients undergoing cardiac surgery

**DOI:** 10.5935/1678-9741.20150042

**Published:** 2015

**Authors:** Paulo Sérgio da Silva, Márcio Portugal Trindade Cartacho, Casimiro Cardoso de Castro, Marcello Fonseca Salgado Filho, Antônio Carlos Aguiar Brandão

**Affiliations:** 1 Hospital Regional Alto Vale (HRAV), Rio do Sul, SC, Brazil.; 2 Hospital Regional Terezinha Gaio Basso and Hospital São Miguel, São Miguel do Oeste, SC, Brazil.; 3 Hospital dos Cajueiros de Luanda, Luanda, Angola.; 4 Universidade Federal de Juiz de Fora (UFJF), Juiz de Fora, MG, Brazil.; 5 Universidade do Vale do Sapucaí (UNIVAS), Pouso Alegre, MG, Brazil.

**Keywords:** Anesthesia, Airway Extubation, Hypertension, Pulmonary, Heart Valve Diseases, Cardiovascular Surgical Procedures

## Abstract

**Objective:**

To evaluate the influence of pulmonary hypertension in the ultra-fast-track anesthesia technique in adult cardiac surgery.

**Methods:**

A retrospective study. They were included 40 patients divided into two groups: GI (without pulmonary hypertension) and GII (with pulmonary hypertension). Based on data obtained by transthoracic echocardiography. We considered as the absence of pulmonary hypertension: a pulmonary artery systolic pressure (sPAP) <36 mmHg, with tricuspid regurgitation velocity <2.8 m/s and no additional echocardiographic signs of PH, and PH as presence: a sPAP >40 mmHg associated with additional echocardiographic signs of PH. It was established as influence of pulmonary hypertension: the impossibility of extubation in the operating room, the increase in the time interval for extubation and reintubation the first 24 hours postoperatively. Univariate and multivariate analyzes were performed when necessary. Considered significant a *P* value <0.05.

**Results:**

The GI was composed of 21 patients and GII for 19. All patients (100%) were extubated in the operating room in a medium time interval of 17.58±8.06 min with a median of 18 min in GII and 17 min in GI. PH did not increase the time interval for extubation (*P*=0.397). It required reintubation of 2 patients in GII (5% of the total), without statistically significant as compared to GI (*P*=0.488).

**Conclusion:**

In this study, pulmonary hypertension did not influence on ultra-fast-track anesthesia in adult cardiac surgery.

## Abstract (pt)

**Objetivo:**

Avaliar a influência da hipertensão pulmonar na técnica anestésica ultra-fast-track em cirurgia cardíaca de adultos.

**Métodos:**

Estudo retrospectivo. Foram incluídos 40 pacientes divididos em dois grupos: GI (sem hipertensão pulmonar) e GII (com hipertensão pulmonar). Com base em dados obtidos por ecocardiograma transtorácico, considerou-se como ausência de hipertensão pulmonar: uma pressão sistólica da artéria pulmonar 36 mmHg, com velocidade de regurgitação tricúspide <2,8 m/s e ausência de sinais ecocardiográficos adicionais de hipertensão pulmonar; e como presença de hipertensão pulmonar: uma PSAP >40 mmHg associada a sinais ecocardiográficos adicionais de hipertensão pulmonar. Foi estabelecida como influência da HP: a impossibilidade de extubação na sala cirúrgica, o aumento no intervalo de tempo para extubação e a necessidade de reintubação nas primeiras 24h de pós-operatório. Foram realizadas análises univariada e multivariada quando necessário. Foi considerado como significativo um valor de *P*<0,05.

**Resultados:**

O GI foi composto por 21 pacientes e o GII por 19. Todos os pacientes (100%) foram extubados na sala cirúrgica em um intervalo de tempo médio de 17,58±8,06 min, com uma mediana de 18 min no GI e 17 min no GII. A hipertensão pulmonar não aumentou o intervalo de tempo para extubação (*P*=0,397). Foi necessária a reintubação de 2 pacientes do GII (5% do total), estatisticamente sem significância em relação ao GI (*P*=0,488). Não houve óbitos durante a internação dos pacientes.

**Conclusão:**

Neste estudo a hipertensão pulmonar não teve influência na técnica anestésica *ultra-fast-track* em cirurgia cardíaca de adultos.

**Table:** 

**Abbreviations, acronyms & symbols**	
Ao	Aortic
CABG	Coronary artery bypass graft
CPB	Cardiopulmonary bypass
CVP	Central venous pressure
DM	Diabetes mellitus
ECG	Electrocardiogram
IAC	Interatrial communication
ICU	Intensive Care Unit
LVEF	Left ventricular ejection fraction
PAVSD	Partial atrioventricular septal defect
PFO	Patent foramen ovale
PH	Pulmonary hypertension
PO	Postoperative
PVR	Pulmonary vascular resistance
RV	Right ventricle
SAH	Systemic arterial hypertension
sPAP	Systolic pulmonary artery pressure
TEA	Thoracic epidural analgesia
UFT	Ultra-fast-track
UFTA	Ultra-fast-track anesthesia

## INTRODUCTION

The anesthetic management of patients undergoing cardiac surgery had as standard anesthetic technique the use of high doses of opioids. With this technique patients were extubated after long hours postoperatively, which caused a longer stay in the intensive care unit (ICU), possible complications and increased morbidity and mortality, generating higher costs to the system health^[1]^.

From the 90s, several studies have shown that the use of anesthetic technique that enabled early extubation after cardiac surgery, is a key factor for early discharge^[1,2]^. Krohn et al.^[3]^, described the first rapid management which has been named fast-track. Anesthetic practice adapted to fast-track uses short-term drug or the long-term low dose, targeting an extubation within 6 to 8 hours (fast-track anesthesia)^[1,2]^. Other studies have shown that early extubation is safe and is associated with lower costs^[4,5]^. This type of management is considered the current standard in various referral centers for cardiac surgery^[2]^.

Walji et al.^[6]^ described a protocol that allowed ultra-early hospital discharge, between the 1^st^ and the 4^th^ postoperative (PO) day, which became known as ultra-fast-track management (UFT). This term was extended later to anesthesia as a reference to an anesthetic technique that allows ultra-early extubation in the operating room (ultra-fast-track anesthesia – UFTA)^[2]^. Several studies have shown the safety of this new type of anesthetic management in adult and pediatric patients^[7-11]^.

Pulmonary hypertension (PH) is common in patients with heart disease, being the echocardiography the most useful noninvasive test for evaluating patients with clinical features of PH^[12]^.

The literature does not indicate whether PH, as a prior disease, affects the UFTA of adult patients undergoing cardiac surgery. Most available studies have been performed in children or limited to reports of a few cases in adults with conflicting results.

The aim of this study was to assess the influence of the previous PH in UFTA of adult patients undergoing cardiac surgery.

## METHODS

This was a retrospective study in which the medical records of patients undergoing cardiac surgery and UFTA were analyzed in the period from January to June 2011, at the Cardiovascular Surgery Service of the Josina Machel Hospital - Luanda/Angola.

Forty patients were included in the study. Patients were divided into two groups: Group 1 (GI) - without PH; Group 2 (GII) - with PH.

Data for determining the presence or absence of PH were obtained by transthoracic echocardiography.

It was considered as the absence of PH: a systolic pulmonary artery pressure (sPAP) <36 mmHg, with tricuspid regurgitant velocity <2.8 m/s and no additional echocardiographic signs of PH^[13,14]^. The presence of PH was considered when: sPAP >40 mmHg associated with additional echocardiographic signs of PH^[13,14]^.

The following patients were excluded from the study: patients with sPAP between 36 and 40 mmHg (patients who would need further investigation to confirm PH)^[14]^, patients under the age of 18 years, who underwent surgery without CPB, with aortic disease or undergoing aortic surgery.

The anesthetic technique used was balanced: intravenous and inhalation. The monitoring was performed with pulse oximetry, cardioscopy (ECG), invasive blood pressure measurement, measurement of central venous pressure (CVP), capnography, measurement of nasopharyngeal temperature and measure of urine volume per urinary catheterization. Serial measurement of arterial blood gases, electrolytes dosage and erythrocyte were performed. The induction of anesthesia was performed with etomidate (0.3 mg/kg), lidocaine (1 mg/kg), fentanyl (2 - 5 µg/kg), remifentanil (0.3 µg/kg/min) and rocuronium (1 mg/kg). Maintenance was performed with isoflurane (1.2%) and remifentanil (0.1 - 0.3 µg/kg/min). Postoperative analgesia was performed with spinal morphine (300 μg) (in patients without contraindications) and dipyrone (30 mg/kg/dose) intravenously. In patients not receiving spinal anesthesia, intravenous doses of dipyrone (30 mg/kg/dose), ketorolac (1 mg/kg) and tramadol (1 mg/kg) were administered. Reversal of neuromuscular blockade was performed in all patients.

We considered as criteria for extubation patients who presented at the end of surgery: 1) hemodynamically stable or under use of low dose vasoactive drug; 2) balanced at the electrolyte and acid-base viewpoint (by arterial blood gases); 3) with adequate analgesia; 4) with enough level of consciousness to the respiratory control; 5) without respiratory complications from the surgical procedure; 6) without major bleeding.

Three factors were assessed to determine if there was PH's influence on UFTA, leading to failure in technique: 1) the impossibility of extubation in the operating room; 2) the extended period of time to extubation; 3) the need for reintubation within 24 hours PO.

Statistical analysis was performed using R software version 3.0.3. To verify the homogeneity of the groups regarding the study variables, the Mann-Whitney test for quantitative variables and the chi-square test and Fisher exact for categorical variables were used. To assess whether there were factors that influence the time interval for extubation we used Log-Linear regression analysis via Quasi-Likelihood, with univariate and multivariate analyzes through the Backward and Forward procedures, and the final regression being for the time extubation called Log-Linear Stepwise. To assess whether there were factors that influence the reintubation the univariate analysis was performed, using for such, Mann-Whitney tests for quantitative variables and the Fisher exact test for categorical variables. At the end of all the analyzes the Statistical significance was defined with "*P*" values less than 5% (*P*<0.05).

This study was approved by the Research Ethics Committee of the Josina Machel Hospital where the written informed consent of the patients was delivered.

## RESULTS

The 40 patients studied had undergone various types of surgery, the vast majority valve surgeries, predominantly surgeries involving the mitral valve. The number of patients distributed between the two groups was very close, and the GI was composed of 21 patients and GII composed of 19 patients. In GII most patients (63.16%) had moderate PH, and the average sPAP in this group was 64.15±17.78 mmHg (minimum sPAP of 44 mmHg and maximum of 121 mmHg) (Tables 1 and 2).

**Table 1:** Types of surgeries performed.

	N	GI (without PH)	GII (with PH)
Mitral valve replacement	19	10	9
Mitral valve replacement + tricuspid plasty	4	1	3
Mitral valve replacement + PFO Correction	1	1	0
Mitral valve replacement + IAC Correction	1	1	0
Aortic valve replacement (AoVR)	6	6	0
Mitral valve replacement + AoVR	2	1	1
Tricuspid valve replacement	1	0	1
IAC Correction	3	0	3
Resection of subaortic membrane	1	1	0
Correction of pulmonary stenosis	1	0	1
Correction of PAVSD	1	0	1
**Total**	**40**	**21**	**19**

PH=pulmonary hypertension; PFO=patent foramen ovale; IAC=interatrial communication; PAVSD=partial atrioventricular septal defect

**Table 2:** sPAP in GII patients.

	sPAP (mmHg)	Medium		SD	Min.		Max.
		64.15		17.78	44		121
			N			%	
Slight PH	36 - 50		3			15.79%	
Moderate PH	51 - 70		12			63.16%	
Severe PH	>70		4			21.05%	

sPAP=systolic pulmonary artery pressure; SD=standard deviation; Min.=minimum; Max.=maximum; PH=pulmonary hypertension

Most patients were female, representing 52% of GI patients and 74% of GII. The EuroSCORE was of low risk in 86% of GI patients and 53% of GII. The average age of patients was 34.9±14.23 years (median 33 years in GI and 36 years in GII), the average weight of 53.40±12.53 kg (median 52 kg in GI and 47 kg in GII) and the average height of 1.62±0.10 meters (median of 1.7 meters in GI and 1.6 meters in GII). No patient had diabetes mellitus (DM) or chronic obstructive pulmonary disease (COPD). Most were not carriers of systemic arterial hypertension (SAH) (77.5%) or had the smoking habit (90%). Left ventricular ejection fraction (LVEF) average was 62±9% (median 60% in both groups). The spinal analgesia was performed in 8 patients (20%). The mean duration of surgery was 156.23±40.97 minutes (median 158 minutes in GI and 150 minutes in GII), the cardiopulmonary bypass (CPB) 56.75±25.32 minutes (median 48 minutes in GI and 51 minutes in GII) and the aortic clamping 41.93±19.22 minutes (median 35 minutes in GI and 38 minutes in GII). Dobutamine has been used on 8 patients (20%) and sodium nitroprusside in 31 patients (77.5%). All patients (100%) were extubated in the operating room and the average time interval for extubation was 17.58±8.06 minutes (median 18 minutes in GI and 17 minutes in GII). It required reintubation of two patients (5%) in the first 24 hours PO. There were no deaths during hospitalization of patients (Tables 3 to 6).

**Table 3:** Characteristics of patients and perioperative - Frequency for categorical variables.

Variables		N	%
Group	GI (without PH)	21	52.5%
	GII (with PH)	19	47.5%
Gender	Female	25	62.5%
	Male	15	37.5%
EuroSCORE	Low	28	70.0%
	Medium	11	27.5%
	High	1	2.5%
DM	No	40	100.0%
	Yes	0	0.0%
SAH	No	31	77.5%
	Yes	9	22.5%
COPD	No	40	100.0%
	Yes	0	0.0%
Smoking	No	36	90.0%
	Yes	4	10.0%
Spinal analgesia	No	32	80.0%
	Yes	8	20.0%
Dobutamine	No	32	80.0%
	Yes	8	20.0%
Sodium nitroprusside	No	9	22.5%
	Yes	31	77.5%
Extubation in the operating room	No	0	0.0%
	Yes	40	100%
Reintubation	No	38	95.0%
	Yes	2	5.0%
Deaths during hospitalization	No	40	100%
	Yes	0	0.0%

PH=pulmonary hypertension; DM=diabetes mellitus; SAH=systemic arterial hypertension; COPD=chronic obstructive pulmonary disease

**Table 5:** Characteristics of patients - Homogeneity of groups.

Variables		Groups				*P*-value
		GI (without PH)		GII (with PH)		
Gender	Female	11	52%	14	74%	0.165
	Male	10	48%	5	26%	
Age (years)		33.0	[22.0; 41.0]	36.0	[21.0; 47.0]	0.654
Weight (kg)		52.0	[46.5; 66.0]	47.0	[43.2 ; 52.5]	0.036
Height (m)		1.7	[1.6; 1.7]	1.6	[1.5; 1.7]	0.026
DM	Yes	0	0%	0	0%	
	No	21	100%	19	100%	
SAH	Yes	4	19%	5	26%	0.712
	No	17	81%	14	74%	
COPD	Yes	0	0%	0	0%	
	No	21	100%	19	100%	
Smoking	Yes	2	10%	2	11%	1.000
	No	19	90%	17	89%	
LVEF (%)		60	[50; 70]	60	[60; 70]	0.849
EuroSCORE	Low	18	86%	10	53%	0.051
	Medium	3	14%	8	42%	
	High	0	0%	1	5%	

For categorical variables were presented absolute and relative frequency and for quantitative variables was presented Q2 [Q1;Q3]. PH=pulmonary hypertension; DM=diabetes mellitus; SAH=systemic arterial hypertension; COPD=chronic obstructive pulmonary disease; LVEF=left ventricular ejection fraction

**Table 6:** Perioperative - Homogeneity of groups.

Variables		Groups				*P*-value
		GI (without PH)		GII (with PH)		
Spinal analgesia	Yes	5	24%	3	16%	0.698
	No	16	76%	16	84%	
Surgery time (min)		158.0	[120.0; 180.0]	150.0	[128.0;170.0]	0.674
CPB time (min)		48.0	[39.0; 63.0]	51.0	[43.5; 63.0]	0.386
Aortic clamping time (min)		35.0	[28.0; 48.0]	38.0	[34.0; 42.5]	0.255
Use of dobutamine	Yes	2	10%	6	32%	0.120
	No	19	90%	13	68%	
Use of sodium nitroprusside	Yes	17	81%	14	74%	0.712
	No	4	19%	5	26%	
Extubation in the operating room	Yes	21	100%	19	100%	
	No	0	0%	0	0%	
∆T extubation (min)		18.0	[10.0; 22.0]	17,0	[13.5; 21.0]	0.776
Reintubation	Yes	0	0%	2	10%	0.488
	No	21	100%	17	90%	
Deaths in hospital	Yes	0	0%	0	0%	
	No	21	100%	19	100%	

For categorical variables were presented absolute and relative frequency and for quantitative variables was presented Q2 [Q1; Q3]. PH=pulmonary hypertension; CPB=cardiopulmonary bypass; ∆T extubation=time interval for extubation (time between turning off the halogenated gas and patient's extubation); min=minutes

The groups were not homogeneous with respect to two variables, the weight and height, and the median for these variables were significantly higher in GI when compared to GII, with a *P* value of 0.036 for weight and 0.026 for height (Table 5).

Since they were not homogeneous groups, to assess the possible variables which influence the time interval for extubation, it was necessary to perform univariate and multivariate analyzes. From the univariate analysis the only factor that could have influenced the time interval for extubation was the height. When performing multivariate analysis, controlled by the factor group (GII), which is the aim of the study, it was observed that the height influences the time interval for extubation in inverse ratio, so that the lower the height, the greater the time interval for extubation (*P*=0.034). Pulmonary hypertension, in turn, had no influence on the time interval for extubation (*P*=0.397) (Table 7).

**Table 7:** ∆T extubation - multivariate regression Log-Linear Stepwise.

	*P* value	exp(β)	CI - 95%
Group = GII	0.397	0.90	[0.70; 1.15]
Height (m)	0.034	0.99	[0.97; 1.00]

In GII there were 2 patients who were reintubated in the first 24 hours postoperatively; however, with no statistical difference when compared to GI (*P*=0.488). No variable influenced the reintubation. This finding was mainly due to the small sample size (Table 8).

**Table 8:** Reintubation.

Variables		No		Yes		*P*-value
Group	GI	21	55%	0	0%	0.488
	GII	17	45%	2	100%	
Gender	Female	24	63%	1	50%	1.000
	Male	14	37%	1	50%	
Age (years)		33	[21.0; 44.0]	29.5	[22.0; 37.0]	0.803
Weight (kg)		50	[45.0; 59.5]	50.5	[35.0; 66.0]	0.733
Height (m)		1.6	[1.5; 1.7]	1.7	[1.5; 1.9]	0.514
SAH	No	29	76.30%	2	100.00%	1.000
	Yes	9	23.70%	0	0.00%	
Smoking	No	34	89.50%	2	100.00%	1.000
	Yes	4	10.50%	0	0.00%	
LVEF (%)		60	[60; 70]	50	[50; 60]	0.162
EuroSCORE	Low	27	71%	1	50%	0.515
	Medium	10	26%	1	50%	
	High	1	3%	0	0%	
Spinal analgesia	No	30	79%	2	100%	1.000
	Yes	8	21%	0	0%	
Surgery time (min)		152.5	[120.0; 176.0]	207,5	[140.0; 275.0]	0.368
CPB time (min)		49.5	[40.0; 63.0]	77	[48.0; 106.0]	0.321
Aortic clamping time (min)		36.5	[30.0; 43.0]	55.5	[28.0; 83.0]	0.756
Dobutamine	No	31	81.60%	1	50.00%	0.364
	Yes	7	18.40%	1	50.00%	
Sodium nitroprusside	No	8	21.10%	1	50.00%	0.404
	Yes	30	78.90%	1	50.00%	
∆T extubation (min)		16.5	[10.0; 22.0]	19.5	[18.0; 21.0]	0.576

For categorical variables were presented absolute and relative frequency and for quantitative variables was presented Q2 [Q1; Q3]. SAH=siystemic arterial hypertension; LVEF=left ventricular ejection fraction; CPB=cardiopulmonary bypass

## DISCUSSION

This study assessed young adults, predominantly female, with few comorbidities, with low to moderate cardiovascular risk, mostly affected by heart valve disease with prevalence of mitral valve disease. Only two patients underwent coronary artery bypass graft (CABG), but these were excluded for not using CPB. The results found in this study was similar to other cardiac surgery services in sub-Saharan Africa in terms of patient characteristics and operated pathologies, where rheumatic fever is highly prevalent, and is a major cause of valve disease with surgical need in young adults^[15-17]^. The coronary diseases are uncommon in this population, probably due to low life expectation.

Borraci et al.^[18]^ analyzing the causes of failure in UFTA in coronary artery bypass graft surgery (CABG), valve or combined, observed that the factors that may predict difficulty of extubation in the operating room in surgery with cardiopulmonary bypass (CPB) are: heart failure, left ventricular dysfunction, emergency surgery, prolonged time of aortic clamping, difficulty to exit CPB and the need for pacemaker use; and in surgeries without CPB: lung disease, obesity, emergency surgery, the need for pacemaker use and hemodynamic instability. Dáyan et al.^[19]^ cited as possible causes of failure of UFTA: female gender, obesity and a history of cardiac failure. Royse et al.^[20]^reported severe pulmonary hypertension as a cause of failure in one of its UFTA pacientes. Vida et al.^[21]^, on the other hand, analyzing children, concluded that PH does not contraindicate early extubation.

Rady et al.^[22]^, in a cohort study of 11,330 patients admitted to the ICU, analyzing the causes of extubation failure (requiring reintubation and mechanical ventilation), cited, among other causes, PH.

All study patients were extubated in the operating room. No single factor led to extubation failure. The sPAP in the GII ranged 44-121 mmHg, and even patients with severe HP were extubated, so that the PH had no influence at the time of extubation.

Most studies with extubation in operating room in adults were performed in patients undergoing off-pump surgery. If we consider the majority of the available studies involving patients undergoing on-pump and off-pump surgery, extubation rate in the operating room varies from 42 to 100%. Some authors cite the time to extubation ranging between 10 and 20 minutes after skin closure, others mention only the patients who were extubated^[3,7-9,18,19]^. Only two studies in the literature involving surgery with CPB in adults showed rate of 100% immediate extubation. The study by Hermmeling et al.^[8]^ with 45 patients who were extubated within 15 minutes of the end of the surgery, in which balanced anesthesia was used with a low dose of fentanyl and maintenance with sevoflurane and the study of Oxelbark et al.^[9]^ with 250 consecutive patients, all of which being extubated within 10 minutes from skin closure. In the latter study total intravenous anesthesia with propofol and remifentanil was used. In both cases thoracic epidural analgesia (TEA) was performed. Neither studies report PH as an aggravating factor. In another study by Hermmeling et al.^[23]^ in patients operated without CPB and TEA, undergoing UFTA, compared the effects of isoflurane and sevoflurane between two groups of patients. There was no difference in terms of myocardial protection, cardiac contractility and hemodynamic stability, but the time to extubation was significantly lower in the group using sevoflurane (10±5 minutes) compared to the group using isoflurane (18±4 minutes).

Extubation rate in our study was 100%, with an average time interval for extubation of 17.58±8.06 min, with a median of 18 min in GI and 17 min in GII - no statistical difference between the two groups. PH had no influence on the time interval for extubation of patients. We believe that the fact that we used as halogenated isoflurane may have been the cause of a slightly longer time for extubation compared to studies by Hermmeling et al.^[8]^ and Oxelbark et al.^[9]^ In the present study TEA was not performed, analgesia was performed with spinal morphine (in 8 patients) associated with venous analgesia or only venous analgesia, and this factor had no influence on UFTA.

The height of the patients, even after multivariate analysis, showed to have had influence on the time interval for extubation, and the lower the height, the greater the time interval for extubation. We did not find in literature studies mentioning short stature as the only factor in the increase of time for extubation.

In the first 24 hours after surgery, 2 patients of GII required reintubation, representing 5% of patients. The reintubation rate presented in the study is within the range reported in the literature in UFTA ranging 0 to 8%^[7-9,18,19,23]^. There was no statistical difference between the two groups. PH and any other factor influenced the reintubation.

When assessing the causes of reintubation of two patients, one was reintubated by CO_2_ retention and another was reintubated due to seizure followed by cardiorespiratory arrest (CPA). The first patient was extubated again one hour later and the second after 24 hours of intubation. It was found in the history of the first patient that he had important area of pulmonary fibrosis due to tuberculosis sequel, frequent pathology in developing countries in Africa. This may have been responsible for respiratory complication of this patient in the PO and likely because of his reintubation. The opening of the left chambers in cardiac surgery predisposes to gas embolism, which we believe have been the cause of the seizure of the latter patient, since it had no history of seizures before surgery. The major causes of reintubation in UFTA cited in the literature are: respiratory depression and bleeding^[18,19]^. The seizure has not been among them. We suggest giving special importance to the history of pulmonary disease in patients eligible for UFTA and the patients carriers of such disease should perhaps be referred under intubation to ICU, and consider the time of heart deaeration (removal of intracardiac air) as a surgical time to be respected, in order to avoid postoperative complications leading to reintubation.

The sPAP of reintubated patients was 48 mmHg in the patient who presented CO_2_ retention and 47 mmHg in the patient reintubated for seizure followed by CPA. We believe that the reintubation of these patients is not related to their PH, but with tuberculous lung of the first patient and postoperative complication of the second one.

Some authors claim that PH is an important risk factor for intraoperative morbidity and mortality^[12]^. In the present study, although the patients have presented some degree of intraoperative morbidity, there were no deaths during surgery or in the postoperative period.

We consider the main limitation of this study the small number of patients, being necessary to the future, to perform studies with a larger population in order to generate a more solid evidence on the subject.

## CONCLUSION

PH had no influence on UFTA in adult heart surgery, because it did not preclude the extubation in the operating room, it did not increase the time interval for extubation and it was not cause of reintubation of patients in the first 24 hours postoperatively. In the population studied pulmonary hypertension had no effect on in-hospital mortality.

**Table:** 

**Authors’ roles & responsibilities**	
PSS	Analysis and/or interpretation of data; final approval of the manuscript; Conception and study design; Implementation of projects and/or experiments; manuscript writing or critical review of its contents
MPTC	Study design; collection, analysis and interpretation of data
CCC	Data collection
MFSF	Analysis and interpretation of data; manuscript writing
ACAB	Manuscript writing

## Figures and Tables

**Table 4:** Characteristics of patients and perioperative - Descriptive measures of quantitative variables.

Variables	Medium	SD	Min.	Q1	Q2	Q3	Máx.
Age (years)	34.90	14.23	18.00	21.00	33.00	43.00	71.00
Weight (kg)	53.40	12.53	35.00	45.00	50.00	59.75	94.00
Height (m)	1.62	0.10	1.35	1.55	1.63	1.69	1.90
LVEF(%)	62.00	9.00	41.00	57.00	64.00	68.00	75.00
Surgery Time (min)	156.23	40.97	80.00	122.00	152.50	178.00	275.00
CPB Time (min)	56.75	25.32	29.00	40.50	49.50	64.00	160.00
Aortic clamping time (min)	41.93	19.22	17.00	29.50	36.50	45.50	115.00
∆T extubation	17.58	8.06	5.00	11.00	17.50	21.50	47.00

Q1=1^st^ quartile; Q2=2^nd^ quartile (median); Q3=3^rd^ quartile; Kg=kilograms; m=meters; min=minutes; LVEF=left ventricular ejection fraction in %; CPB=cardiopulmonary bypass; ∆T extubation=time interval for extubation (time between turning off the halogenated gas and patient's extubation)
